# Supplementary material for: Bioengineering a plant NLR immune receptor with a robust binding interface toward a conserved fungal pathogen effector
Source: Proc Natl Acad Sci U S A. 2024 Jul 5;121(28):e2402872121. doi: 10.1073/pnas.2402872121 (PMC11252911; doi:10.1073/pnas.2402872121)
Supplement: Supplementary file 1 — Appendix 01 (PDF) [file pnas.2402872121.sapp.pdf]

PNAS

Bioengineering a plant NLR immune receptor with a robust binding interface towards a conserved fungal pathogen effector

Rafał Zdrzałek[1], Yuxuan Xi[1], Thorsten Langner[2,3], Adam R. Bentham[1,2], Yohann Petit-Houdenot[2,4], Juan Carlos De la Concepcion[1,2,5], Adeline Harant[2], Motoki Shimizu[6], Vincent Were[2], Nicholas J. Talbot[2], Ryohei Terauchi[6,7], Sophien Kamoun[2], Mark J. Banfield[1#]

[1] Department of Biochemistry and Metabolism, John Innes Centre, Norwich Research Park, Norwich, NR4 7UH, UK

[2] The Sainsbury Laboratory, University of East Anglia, Norwich Research Park, NR4 7UH, Norwich, UK

[3] Current address: Department of Molecular Biology, Max-Planck-Institute for Biology, 72076, Tuebingen, Germany

[4] Current address: Université Paris-Saclay, INRAE, UR BIOGER, 91120, Palaiseau, France

[5] Current address: Gregor Mendel Institute of Molecular Plant Biology, Austrian Academy of Sciences, Vienna, 1030, Austria

[6] Division of Genomics and Breeding, Iwate Biotechnology Research Center, Iwate, 024-0003, Japan

[7] Laboratory of Crop Evolution, Graduate School of Agriculture, Kyoto University, Kyoto, 606-8501, Japan

Mark J. Banfield

Email: mark.banfield@jic.ac.uk

This PDF file includes:

Figures S1 to S14

Tables S1 to S3

SI References

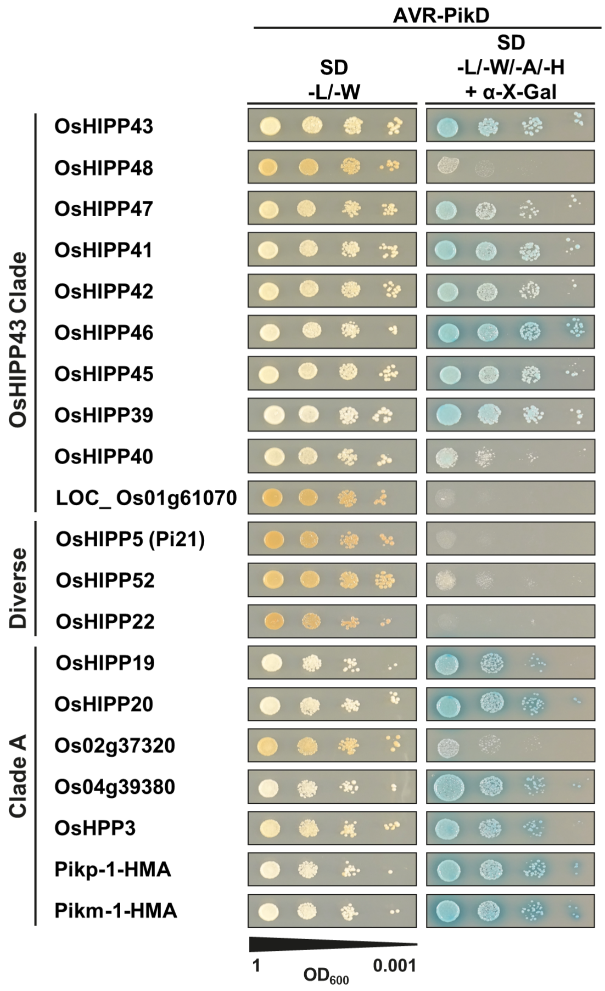

Fig. S1. AVR-PikD interacts with different HMA domains in Yeast 2-hybrid screen. Yeast 2-hybrid shows AVR-PikD interacts with various HMA proteins across the HMA phylogeny. Blue colonies on selective medium (-L/-W/-A/-H + X-α-gal) indicate positive interaction.

```
Pwl2      GGGWTNKQFYNDKGEREGSISIRKGSEGDFNYGPSYPGGFDRMVRVHENNGNIRGMPPGY 81
Pwl2-2    GGGWTNKQFYNDKGEREGSISIRKGSEGDFNYGPSYPGGFDRMVRVHENNGNIRGMPPGY 81
Pwl2-3    GGGWTHKQFYNDKGEREGSISIRKGSEGDFNYGPSYPGGFDRMVRVHENNGNIRGMPPGY 81
          *****:******************************************************

Pwl2      SLGPDHQEDKSDRQYYNRHGYHVGDGPAEYGNHGGGQWGDGYYGPPGEFTHEHREQREEG 141
Pwl2-2    SLGPDHQENKSDRQYYNRHGYHVGDGPAEYGNHGGGQWGDGYYGPPGEFTHEHREQREEG 141
Pwl2-3    SLGPDHQQDQIDRQYYNRHGYHVGDGPAEYGNHGGGQWGDGYYGPPGEFTHEHREQREEG 141
          *******::: *************************************************

Pwl2      CNIM 145
Pwl2-2    CNIM 145
Pwl2-3    CNIM 145
          ****
```

Fig. S2. Sequence alignment of Pwl2 allelic variants identifies five polymorphic residues, with four clustered together. Alignment performed using Clustal Omega (https://www.ebi.ac.uk/Tools/msa/clustalo/). Signal peptides were removed from the alignment. Red boxes indicate positions of key residues involved binding OsHIPP43 as defined in the crystal structure of the complex.

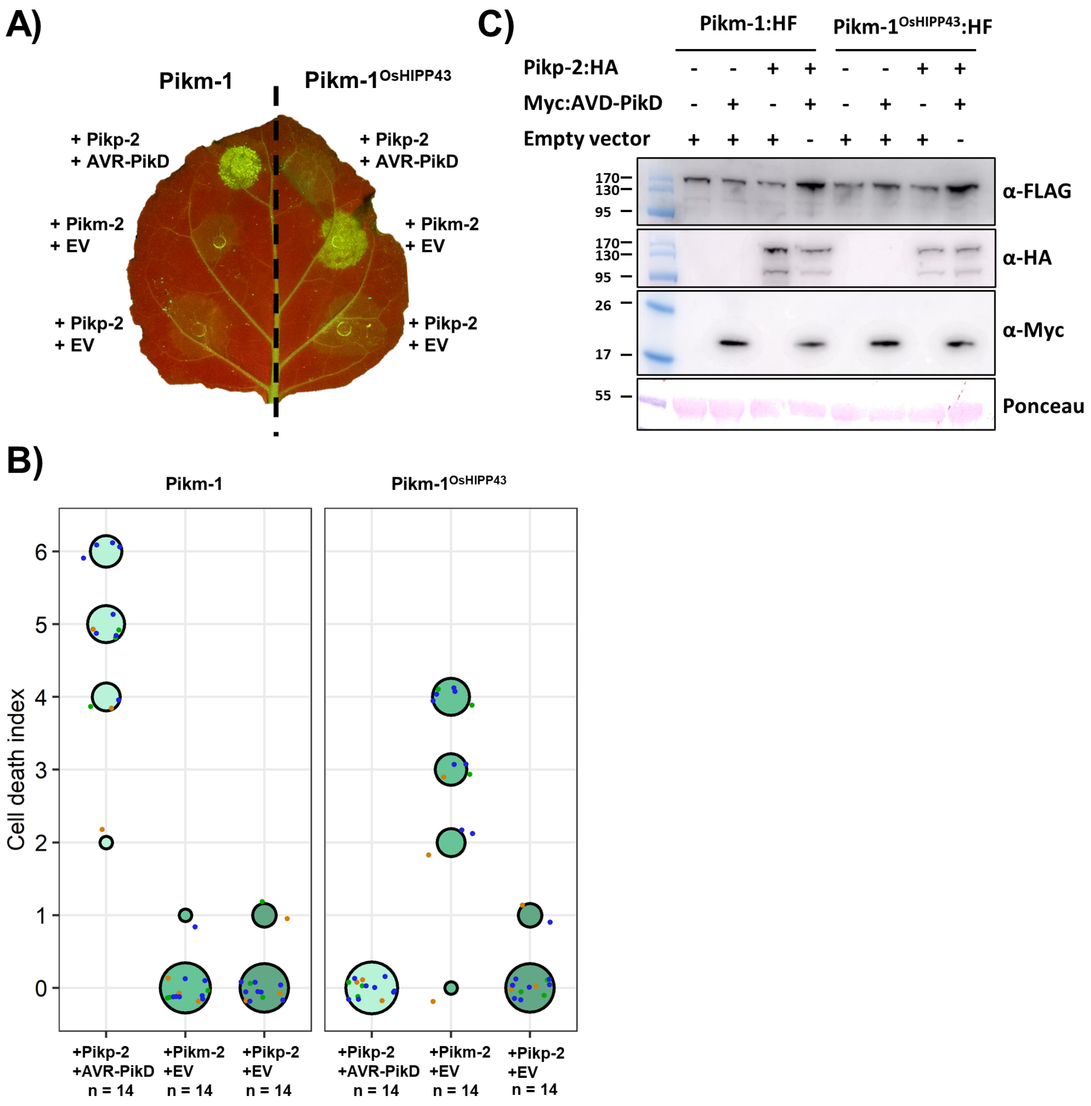

Fig. S3. Auto-activity of Pikm-1^{OsHIPP43} with Pikm-2 can be alleviated by mismatching with Pikp-2. A) Cell death assays showing the chimeric Pikm-1^{OsHIPP43} receptor is auto-active when co-expressed with Pikm-2 in *N. benthamiana*, but not Pikp-2. Leaves were imaged under UV light, allowing visualisation of cell death responses as green fluorescence. **B)** Quantification of the cell death assay presented in **A)** . Each dot represents a single scoring point in the assay**.** All dots are randomly scattered around the cell death score for visualisation (size of the circle at given score is proportional to the number of dots within). The colour of each dot reflects independent biological replicates. "n" indicates the total number of individual scores for each sample. **C)** Western blot confirming that Pikm-1 and Pikm-1^{OsHIPP43} accumulate to similar level in presence and absence of the helper Pikp-2 and effector AVR-PikD. Proteins were transiently expressed in *N. benthamiana* via agroinfiltration and visualised on western blot with relevant antibodies. Ponceau staining was used to demonstrate even protein loading. The amount of agrobacteria used for Empty Vector was adjusted for each sample to give a final OD_{600} of 1.5.

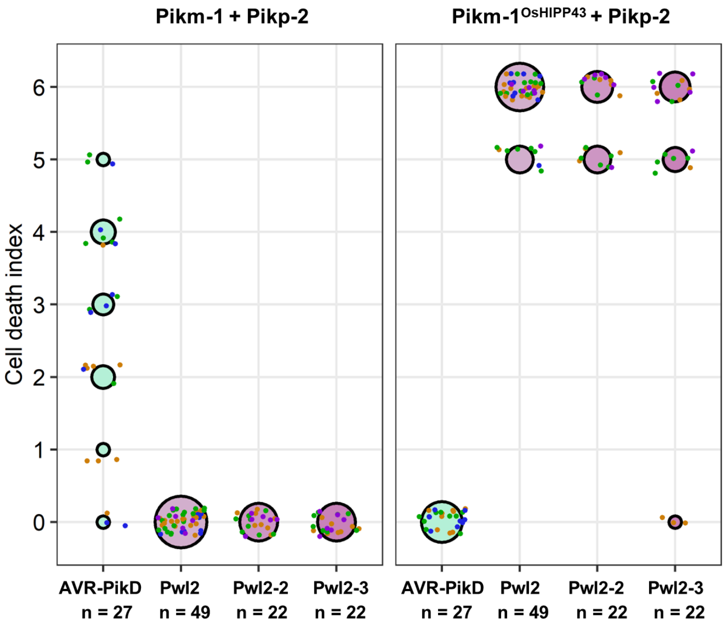

Fig. S4. Quantification of cell death assays presented in Fig. 2. Each dot represents a single scoring point in the assay**.** All dots are randomly scattered around the cell death score for visualisation (size of the circle at given score is proportional to the number of dots within). The colour of each dot reflects independent biological replicates. “n” indicates the total number of individual scores for each sample.

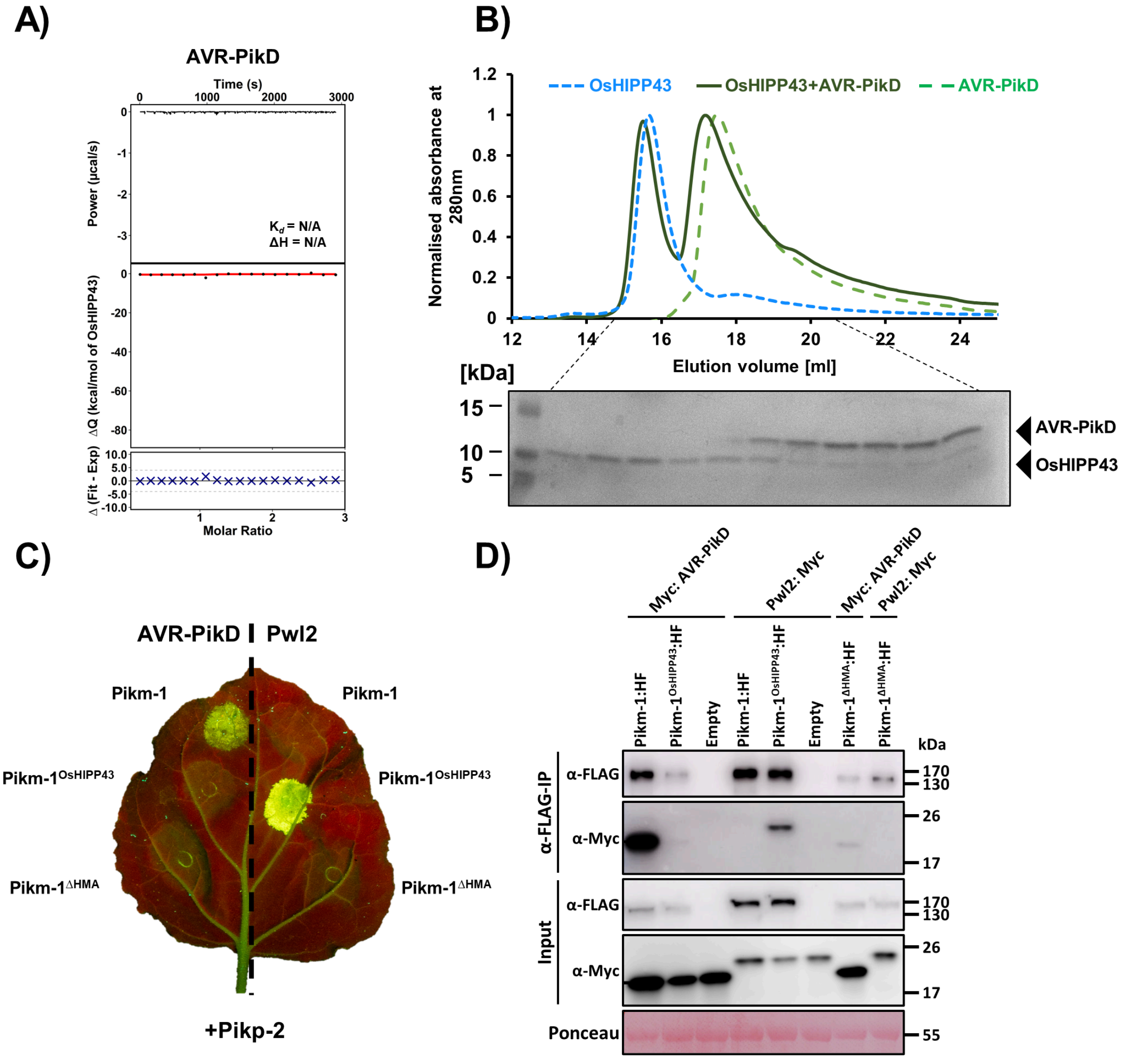

Fig. S5. AVR-PikD does not interact with OsHIPP43 in vitro and is not recognised by the Pikm-1^{OsHIPP43}/Pikp-2 receptor, but can interact with Pikm-1^{OsHIPP43} in planta. A) Titration of AVR-PikD with OsHIPP43 in ITC experiments shows a lack of binding between the proteins. **Top panel-** Representative raw isotherm showing heat exchange upon the series of injections of OsHIPP43 into the cell containing the effector. **Middle panel-** Integrated peaks from technical replicates and global fit to a single site binding model as calculated using AFFINImeter. **Bottom panel-** Difference between predicted value of measurement (by global fit) and actual measurement, as calculated using AFFINImeter. **B)** Following pre-incubation, AVR-PikD and OsHIPP43 elute as distinct peaks (equivalent to individual proteins) on analytical size-exclusion chromatography, supporting the lack of binding observed by ITC. **C)** AVR-PikD is not recognised by the chimeric Pikm-1^{OsHIPP43}/Pikp-2 receptor, nor by the Pikm-1$^{\Delta HMA}$ receptor in the cell death assay. Leaves were imaged under UV light, allowing visualisation of the cell death responses as green fluorescence. **D)** Pwl2 specifically interacts with the chimeric Pikm-1^{OsHIPP43} NLR in co-IP experiments, and AVR-PikD can non-specifically

interact with chimeric Pikm-1 receptors, independent of the HMA region. All proteins were transiently expressed in *N. benthamiana* via agroinfiltration. **Upper panel-** Anti-FLAG immunoprecipitation (αFLAG-IP) was followed by Western Blot detection with relevant antibodies. **Lower panel-** Input confirms presence of all proteins prior to immunoprecipitation. Ponceau staining was used to demonstrate even protein loading.

```
Pwl1    -RKWTNKVIYNDKGPREGSISIRKGAEGDFNCGPGYPGGPDRMVRVHEDNGNIRGMPPGY 82
Pwl2    GGGWTNKQFYNDKGEREGSISIRKGSEGDFNYGPSYPGGPDRMVRVHENNGNIRGMPPGY 81
Pwl3    GRKWLNKKLWDANGQSAGSVSIVKGGQGSINTDTGP-ITAEGSYDIYERNGKIEGGPPGY 80
Pwl4    GRKWFNKKIYDENGESAGSLSVVKGGSGYINIGPSAPGQRDRLVEFRESGGKIQGGPPGY 81
           * ** ::: :*   **:*: **..* :* . .     :    . * .*:*.* ****

Pwl1    RLGPDDKEDKGDNQYYSRNGYHVGDGPAEYQ--NHGGGQ-----WGDGYYGPPGQITNQH 135
Pwl2    SLGPDHQEDKSDRQYYNRHGYHVGDGPAEYG--NHGGGQ-----WGDGYYGPPGEFTHEH 134
Pwl3    KYTEDRYEDRKDDRYYNTHGITSAMDQPNMEIMEVGIGAMDTMVLQGSLYRPANTNSE-- 138
Pwl4    RYTSDHEEDQRDNRYYNTHGYHVGDGPAEYG--DHGGGH-----WGDGYYGPPGEFVKTS 134
            *  **: * :**. :*   . .  :    : * *        .. * * .   .

Pwl1    GKRQGDQGCHIM 147
Pwl2    RE-QREEGCNIM 145
Pwl3    ------------ 138
Pwl4    EYED-------- 138
```

Fig. S6. Pwl variants share between 41 and 79% sequence identity. Sequence alignment of the Pwl family effectors, revealing extensive sequence diversity. Alignment performed using Clustal Omega (https://www.ebi.ac.uk/Tools/msa/clustalo/). Signal peptides were removed from the alignment. Red boxes indicate positions of key residues involved binding OsHIPP43 as defined in the crystal structure of the complex.

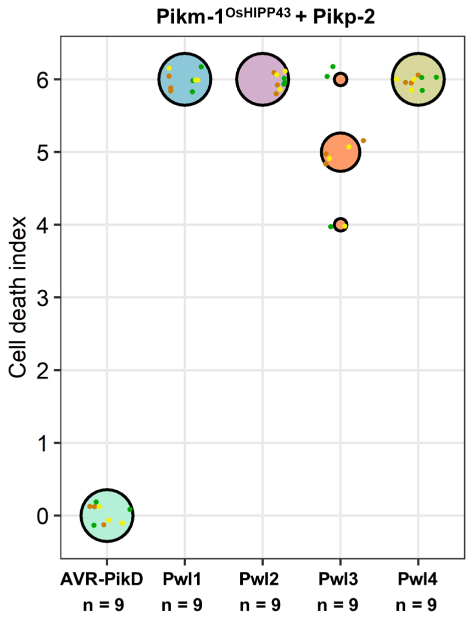

Fig. S7. Quantification of cell death assays presented in Fig. 3. Each dot represents a single scoring point in the assay**.** All dots are randomly scattered around the cell death score for visualisation (size of the circle at given score is proportional to the number of dots within). The colour of each dot reflects independent biological replicates. "n" indicates the total number of individual scores for each sample.

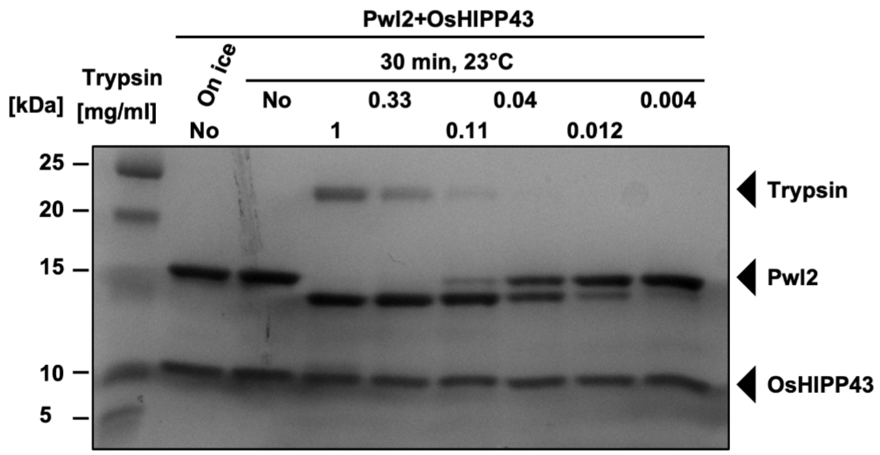

Fig. S8. Partial tryptic digest of the purified Pwl2/OsHIPP43 complex. At higher concentrations of trypsin, Pwl2 is cleaved to yield a smaller, stable fragment while OsHIPP43 remains intact.

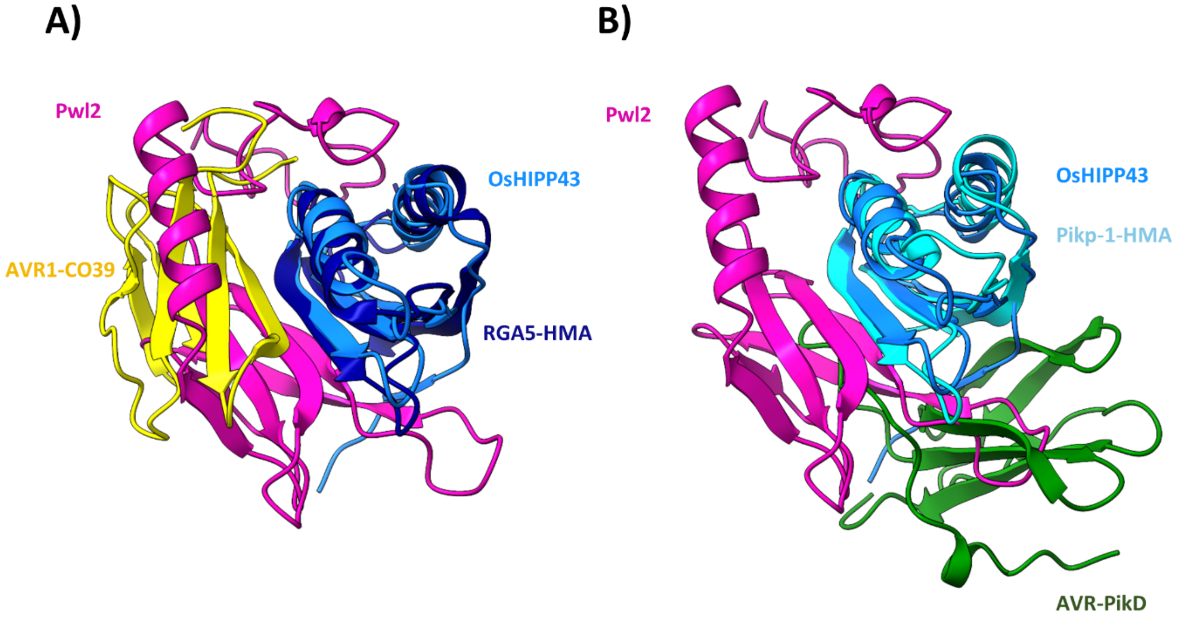

Fig. S9. Comparison of the interaction interfaces between Pwl2/OsHIPP43 and selected characterised MAX effector/HMA complexes. A) Pwl2 (pink) / OsHIPP43 (blue) and AVR1-CO39 (yellow) / RGA5-HMA (dark blue) (PDB: 5ZNG). **B)** Pwl2 (pink) / OsHIPP43 (blue) and AVR-PikD (green) / Pikp-HMA (light blue) (PDB: 6G10). The structures were overlaid using ChimeraX (1).

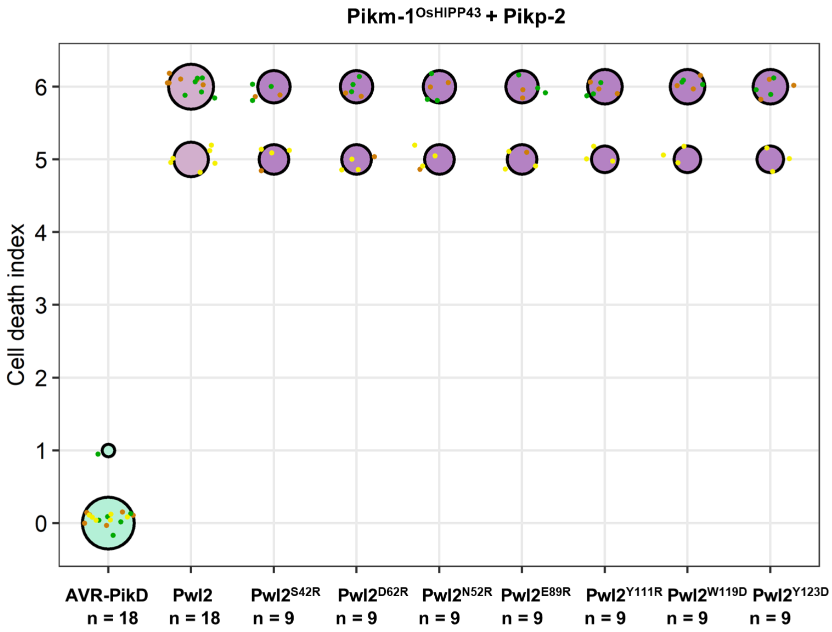

Fig. S10. Quantification of the cell death assays presented in Fig. 5A. Each dot represents a single scoring point in the assay**.** All dots are randomly scattered around the cell death score for visualisation (size of the circle at given score is proportional to the number of dots within). The colour of each dot reflects independent biological replicates. "n" indicates the total number of individual scores for each sample.

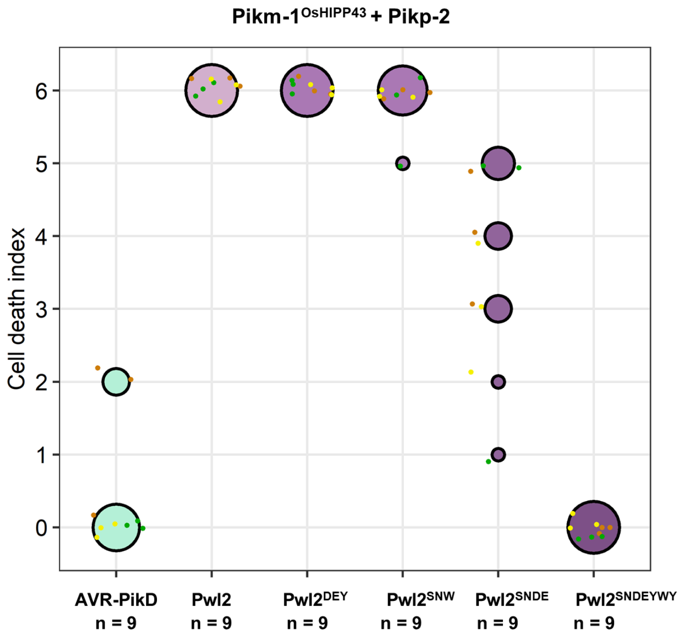

Fig. S11. Quantification of the cell death assays presented in Fig. 5B. Each dot represents a single scoring point in the assay**.** All dots are randomly scattered around the cell death score for visualisation (size of the circle at given score is proportional to the number of dots within). The colour of each dot reflects independent biological replicates. "n" indicates the total number of individual scores for each sample.

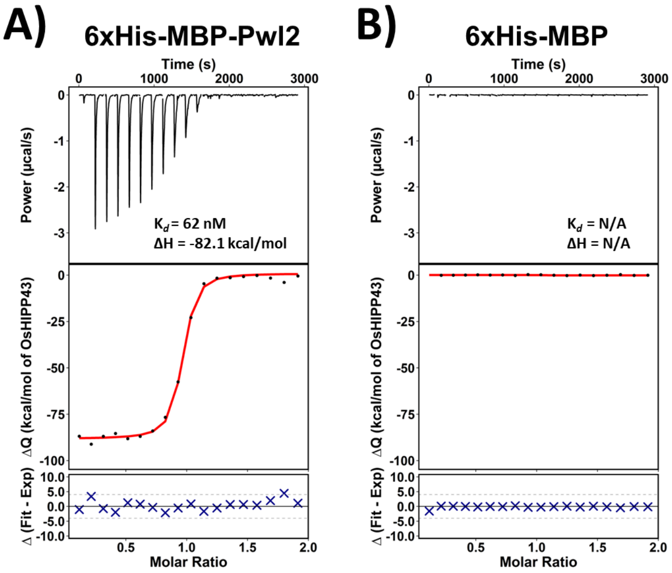

Fig. S12. Presence of the 6xHis-MBP-tag does not interfere with the Pwl2/OsHIPP43 interaction measurements recorded using ITC. A) The affinity of 6xHis-MBP-Pwl2 with OsHIPP43 is comparable to Pwl2 alone. **B)** 6xHis-MBP alone does not show affinity to OsHIPP43. **Top panels-** Representative raw isotherm showing heat exchange upon the series of injections of the OsHIPP43 into the cell containing the effector. **Middle panels-** Integrated peaks from the technical replicates and global fit to a single site binding model as calculated using AFFINImeter. **Bottom panels-** Difference between predicted value of measurement (by global fit) and actual measurement as calculated using AFFINImeter.

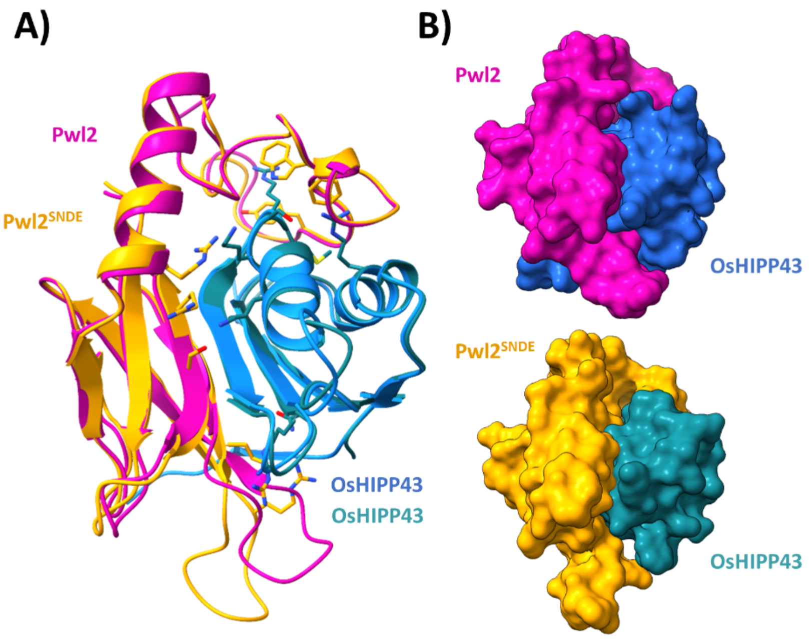

Fig. S13. Crystal structure of the Pwl2^{SNDE}/OsHIPP43 complex reveals how the mutations are accommodated at the interface. A) Superposition of the Pwl2 (pink) / OsHIPP43 (blue) and Pwl2^{SNDE} (orange) / OsHIPP43 (green) complexes, with mutated residues depicted as sticks on the Pwl2^{SNDE}/OsHIPP43 structure. **B)** Side-by-side comparison of surface representation of the two complexes reveals how the introduced mutations in Pwl2^{SNDE} affect the OsHIPP43 interaction interface globally. The structures were overlaid using ChimeraX (1).

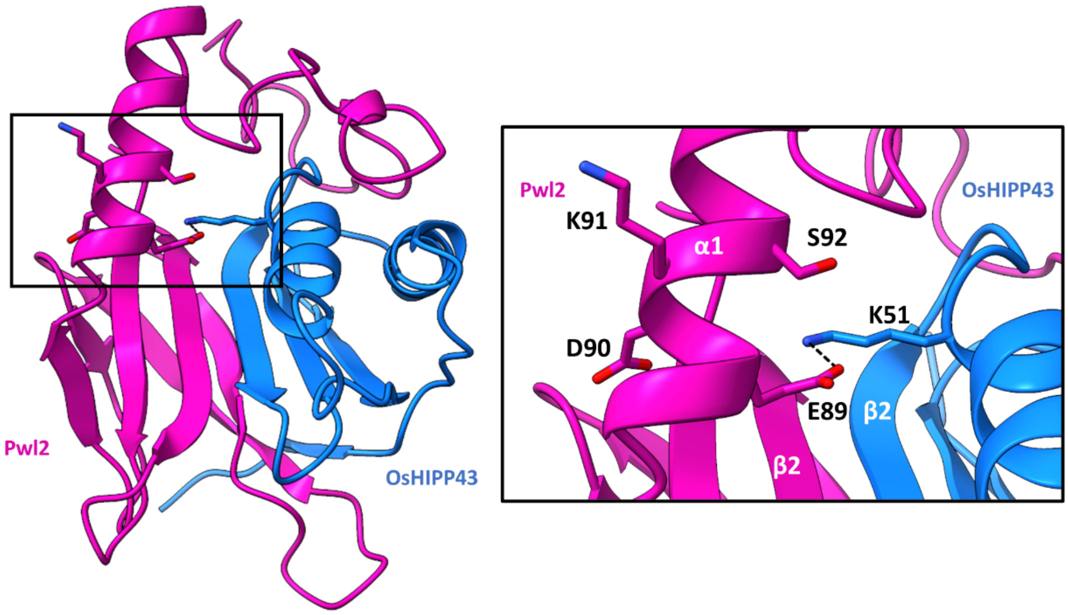

Fig. S14. The polymorphic region of Pwl2 is not buried at an interface with OsHIPP43. Residues Glu-89, Asp-90, Lys-91 and Ser-92 are polymorphic between Pwl2 alleles. Close-up view of the polymorphic region within the Pwl2 (pink) / OsHIPP43 (blue) crystal structure shows that only Glu-89 residue is involved in OsHIPP43 (blue) binding. Side chains shown in stick representation. α-helices and β-strands are labelled, and amino acids labelled with single letter codes. Hydrogen bonds are depicted as black dashes between atoms.

Table S1. Percent identity matrix of HMA domains belonging to the OsHIPP43 clade

	OsHIPP41	OsHIPP42	OsHIPP43	OsHIPP46	OsHIPP45	OsHIPP48	OsHIPP47
**OsHIPP41**	100.00	72.94	57.14	56.79	53.75	43.84	44.87
**OsHIPP42**	72.94	100.00	59.30	61.45	57.50	42.47	41.77
**OsHIPP43**	57.14	59.30	100.00	56.63	60.00	32.88	39.24
**OsHIPP46**	56.79	61.45	56.63	100.00	74.03	40.28	43.59
**OsHIPP45**	53.75	57.50	60.00	74.03	100.00	42.47	41.89
**OsHIPP48**	43.84	42.47	32.88	40.28	42.47	100.00	55.88
**OsHIPP47**	44.87	41.77	39.24	43.59	41.89	55.88	100.00

Table includes OsHIPP43 and its six closest protein family members.

Table S2. ***X*-ray data collection statistics**

	Pwl2/OsHIPP43	Pwl2/OsHIPP43	$Pwl2^{SNDE}$/OsHIPP43
	Native	S-SAD	Native
Wavelength (Å)	0.9795	1.90	0.9795
Space group	$P\ 6_1\ 2\ 2$	$P\ 6_1\ 2\ 2$	$P\ 2_1\ 2_1\ 2_1$
Cell dimensions	63.16 63.16 198.21	63.30 63.30 198.47	66.38 66.80 81.90
a, *b*, *c* (Å)			
Resolution (Å)*	54.70-1.80 (1.84-1.80)	54.82-2.80 (2.95-2.80)	47.09-2.50 (2.60-2.50)
R_{meas} (%)	8.7 (169.8)	13.5 (43.3)	29.6 (139.7)
$I/\sigma I$	31.1 (2.9)	70.5 (28.3)	6.6 (1.9)
Completeness (%)			
Overall	100.0 (100.0)	100.0 (99.8)	100.0 (100.0)
Anomalous	100.0 (100.0)	100.0 (100.0)	100.0 (100.0)
Unique reflections	22749 (1291)	6420 (888)	13157 (1454)
Redundancy			
Overall	36.9 (38.4)	295.3 (230.7)	13.3 (13.9)
Anomalous	20.3 (20.5)	173.7 (129.2)	7.2 (7.3)
$CC^{(1/2)}$ (%)	100.0 (91.5)	99.8 (99.8)	99.2 (76.5)

*The highest resolution shell is shown in parenthesis.

Table S3. Refinement and model validation statistics.

	Pwl2/OsHIPP43	Pwl2^{SNDE}/OsHIPP43
Resolution (Å)	54.76-1.8 (1.85 – 1.80)	47.13-2.50 (2.57-2.50)
R_{work}/R_{free} (%)	18.8 (25.1) / 21.5 (29.5)	21.6 (27.0) / 28.6 (35.0)
No. atoms		
Protein	1473	2847
Ligand	175	43
B-factors		
Protein	43.64	41.88
Ligand	46.00	31.24
R.m.s deviations		
Bond lengths (Å)	0.009	0.007
Bond angles (º)	1.62	1.73
Ramachandran plot (%)**		
Favoured	97.3	97.2
Allowed	2.7	2.5
Outliers	0.0	0.3
MolProbity Score	1.3	2.0

*The highest resolution shell is shown in parenthesis.

**As calculated by MolProbity

SI References

1. E. F. Pettersen, *et al.*, UCSF ChimeraX: Structure visualization for researchers, educators, and developers. *Protein Sci. Publ. Protein Soc.* **30**, 70–82 (2021).
